# Association of the Hermansky–Pudlak syndrome type 4 (*HPS4*) gene variants with cognitive function in patients with schizophrenia and healthy subjects

**DOI:** 10.1186/1471-244X-13-276

**Published:** 2013-10-30

**Authors:** Go Kuratomi, Atsushi Saito, Yuji Ozeki, Takashi Watanabe, Kumiko Fujii, Kazutaka Shimoda, Toshihiko Inukai, Harunobu Mori, Kenichi Ohmori, Kazufumi Akiyama

**Affiliations:** 1 Department of Biological Psychiatry and Neuroscience, Dokkyo Medical University School of Medicine, 880 Kitakobayashi, Mibu, Tochigi 3210293, Japan; 2 Department of Psychiatry, Dokkyo Medical University School of Medicine, 880 Kitakobayashi, Mibu, Tochigi 3210293, Japan; 3 Department of Internal Medicine (Endocrinology, Metabolism, and Hematology), Dokkyo Medical University Koshigaya Hospital, 2-1-50 Minamikoshigaya, Koshigaya, Saitama 3438555, Japan; 4 Mori Hospital, 419 Iidamachi, Utsunomiya, Tochigi 3210347, Japan; 5 Takizawa Hospital, 2-29 Hanabusahoncho Utsunomiya, Tochigi 3200828, Japan

**Keywords:** HPS4, Cognition, Working memory, Executive function, BACS, rs9608491, rs713998

## Abstract

**Background:**

The Hermansky–Pudlak Syndrome Type 4 (*HPS4*) gene, which encodes a subunit protein of the biogenesis of lysosome-related organelles complex (BLOC)-3, which is involved in late endosomal trafficking, is associated with schizophrenia; however, its clinical relevance in schizophrenia remains unknown. The purpose of the present study was to investigate whether *HPS4* is associated with cognitive functions in patients with schizophrenia and healthy controls and with the clinical profiles of patients with schizophrenia.

**Methods:**

We investigated the association of variants of *HPS4* with clinical symptoms and cognitive function in Japanese patients with schizophrenia (n = 240) and age-matched healthy control subjects (n = 240) with single nucleotide polymorphisms (SNP)- or haplotype-based linear regression. We analyzed five tagging SNPs (rs4822724, rs61276843, rs9608491, rs713998, and rs2014410) of *HPS4* and 2–5 locus haplotypes of these five SNPs. The cognitive functions of patients and healthy subjects were evaluated with the Brief Assessment of Cognition in Schizophrenia, Japanese-language version, and the patients were assessed for their symptomatology with the Positive and Negative Symptom Scale (PANSS).

**Results:**

In patients with schizophrenia, rs713998 was significantly associated with executive function under the dominant genetic model (*P* = 0.0073). In healthy subjects, there was a significant association between working memory and two individual SNPs under the recessive model (rs9608491: *P* = 0.001; rs713998: *P* = 0.0065) and two haplotypes (rs9608491-713998: *P* = 0.0025; rs61276843-9608491-713998: *P* = 0.0064). No significant association was found between *HPS4* SNPs and PANSS scores or premorbid IQ, as measured by the Japanese version of the National Adult Reading Test.

**Conclusions:**

These findings suggested the involvement of *HPS4* in the working memory of healthy subjects and in the executive function deficits in schizophrenia.

## Background

Cognitive impairments are a core feature of schizophrenia and a major determinant of functional outcome [1]. Cognitive domains that are particularly impaired in patients with chronic schizophrenia are verbal memory, working memory, motor speed, verbal fluency, attention, and executive function [2,3]. These cognitive domains, specifically working memory and executive function, are heritable traits that have a potential association with genetic variants [4,5]. Therefore, these traits are useful as an intermediate phenotype to investigate the functional association of susceptibility genes for schizophrenia [6,7].

22q12 is one of the regions that show high logarithm of the odds ratio scores in schizophrenia [8-10]. The Hermansky–Pudlak Syndrome Type 4 (*HPS4*) gene (OMIM: 606682) maps to human chromosome 22q12.1, and it consists of 14 exons that span approximately 32 kb of genomic DNA encoding an intracellular trafficking-related cytoplasmic protein named HPS4 [11,12]. The human HPS4 has 708 amino acids and a predicted molecular mass of 77 kDa and makes up BLOC-3 jointly with HPS1 as a 1:1 heterodimer [13-15]. Hermansky–Pudlak Syndrome (HPS; MIM: 203300) is a group of rare autosomal recessive diseases characterized by clinical symptoms, such as oculocutaneous albinism and bleeding diathesis, and is caused by defective biogenesis of lysosome-related organelles, such as melanosomes and platelet-dense granules [16]. In our previous case–control association study, haplotypes that were composed of five tagging single-nucleotide polymorphisms (SNPs) of *HPS4* (rs4822724, rs61276843, rs9608491, rs713998, and rs2014410) were found to be significantly associated with schizophrenia, a finding that was revealed by a sliding window approach for 2–5 locus haplotypes, including rs9608491 as an essential SNP [17]. In addition, we have reported that two Japanese siblings who suffered from the comorbidity of major mental disorders, schizophrenia and major depression, and HPS harbored a nonsense mutation in *HPS4*[17].

HPS is caused by a mutation in one of the genes named *HPS1* to *HPS9*, which encode subunit proteins that consist of multiprotein complexes, the adaptor protein complex-3 (AP-3), and the biogenesis of lysosome-related organelles complex (BLOC)-1, 2, and 3 [18,19]. BLOC-1, BLOC-2, and AP-3 interact to play a pivotal role in sorting and trafficking membrane proteins on early endosomes [20-24]. Dysbindin, which is encoded by the dystrobrevin binding protein 1 (*DTNBP1*) gene (also known as *HPS7*), is one of the subunit proteins of BLOC-1 [25]. Several lines of evidence from linkage, association, and postmortem brain studies have indicated an association between *DTNBP1* and schizophrenia [26]. Genetic variants and haplotypes of *DTNBP1* have frequently been reported to be associated with a number of cognitive functions, including verbal, visual, and general memory [27-30], attention [31,32], and executive function [30,32], in patients with schizophrenia and/or healthy controls. These genetic studies on *DTNBP1*[26-32] led to several research studies on identification of binding partners for dysbindin [21-24]. By contrast, BLOC-3, which comprises HPS1 and HPS4, modulates the intracellular movement and distribution of late endosomes and lysosome-related organelles in cells [14,15,33]. Although the precise function of BLOC-3 remains to be clarified, the interaction between BLOC-3 and Rab GTPases has recently been found [34,35].

In order to investigate the association between putative candidate genes and cognitive impairments in schizophrenia, a portable instrument with a high reliability and validity is required to assess the cognition that is specifically impaired in patients with schizophrenia. Most of the neurocognitive assessment batteries that have previously been used in schizophrenia studies involve detailed assessments of the entire profile of neuropsychological function, including normal functioning, but require lengthy and complex procedures. The Brief Assessment of Cognition in Schizophrenia (BACS) was developed as a neurocognitive battery that enables brief administration (about 40 min) and portability and has high reliability for specifically assessing the domains of cognitive function that are consistently impaired in schizophrenia, including verbal memory, working memory, motor speed, verbal fluency, attention, and executive function [3,36]. The Japanese-language version of BACS (BACS-J) has been developed for the assessment of cognition in Japanese patients with schizophrenia, and its reliability and validity have been confirmed [37].

To the best of our knowledge, *HPS4* has not been reported in relation to cognitive functions in patients with schizophrenia or healthy controls. The aim of the present study was to investigate whether *HPS4* is associated with cognitive functions in patients with schizophrenia and healthy controls and with the clinical profiles of patients with schizophrenia. We initially tested the association of five tagging SNPs in *HPS4* with the clinical profiles and cognitive domains as measured by BACS-J in Japanese patients with schizophrenia, and the association of *HPS4* SNPs with cognitive domains was also tested in healthy controls. In addition, we examined the association of *HPS4* haplotypes and cognitive domains by using a sliding window approach in patients with schizophrenia and healthy controls.

## Methods

### Subjects

This study included 240 patients with schizophrenia (139 males/101 females; age = 48.1 ± 12.4, mean ± standard deviation) and 240 unrelated healthy controls (143 males/97 females; age = 48.0 ± 13.0) who were group-matched for age and sex. The subjects in both groups were aged 21–69 years and were recruited from the Kanto area in Japan and included a substantial portion of the subjects who were investigated in our previous genetic study [17]. The patients with schizophrenia were recruited from the Dokkyo Medical University School of Medicine Hospital and affiliated hospitals, and all of them met the Diagnostic and Statistical Manual of Mental Disorders (DSM-IV) criteria [38] for a diagnosis of schizophrenia. The onset and characteristic clinical course of schizophrenia were confirmed by examination of the medical chart. Only the patients with stable disease prior to the neurocognitive assessments were included. There were no specific medication criteria for exclusion of the patient group with respect to the administration of antipsychotics, anticholinergic drugs, and benzodiazepines. The healthy controls were unrelated volunteers who were recruited mainly from nonprofessional university/hospital staff, and they were screened for the absence of DSM-IV axis-I disorders. Across both groups, we excluded subjects with a history of neurological disorders, significant head injury, or significant drug or alcohol abuse. The objective of the present study was clearly explained to the participants, and written informed consent was obtained from all subjects in accordance with the Declaration of Helsinki [39]. The study was formally approved by the Institutional Review Board of the Ethical Committee of Dokkyo Medical University School of Medicine.

### Genotyping

We focused on genotyping five tagging SNPs (rs4822724, rs61276843, rs9608491, rs713998, and rs2014410) in *HPS4* because all the haplotypes are composed of these tagging SNPs, with rs9608491 being included as an essential SNP in the sliding window analysis of a window size of 2–5 SNPs, and these have been found to be associated with schizophrenia in our previous case–control study [17]. Genomic DNA extraction and genotyping from the peripheral blood samples that were collected from the total subjects were performed as previously described [17]. In brief, we extracted genomic DNA from the peripheral blood samples with a QIAamp DNA Blood Maxi Kit (QIAGEN Inc., Valencia, CA). Two SNPs (rs4822724 and rs9608491) were genotyped with a TaqMan® assay (Assay ID: C___2490761_10 and C__11747810_10, respectively, Applied Biosystems, Foster City, CA) with a TP800 Dice Real Time System (TaKaRa Bio Inc., Otsu, Japan). The other three SNPs (rs61276843, rs713998, and rs2014410) were genotyped with direct DNA sequencing of polymerase chain reaction products with specific primer pairs, as previously reported [17].

### Clinical assessment

Smoking status and education years were self-reported by the subjects. Information on the year of disease onset was collected by chart review and double-checked by the patient. Information regarding psychopharmacological medication was collected by chart review. The dosages of individual antipsychotics were gauged based on the equivalent milligram dosage of haloperidol, and the information was summed for each patient and those of anticholinergic drugs were gauged based on the dosage of biperiden. The prescription status of anxiolytics/hypnotics was evaluated as a categorical variable. Patients were assessed according to their clinical rating of symptomatology of schizophrenia using the Positive and Negative Symptom Scale (PANSS) [40].

### Neurocognitive assessment

The cognitive functions were evaluated in the subjects with version A of BACS-J [37]. The BACS-J cognitive battery consists of the following six subtests that target unique domains of cognition: list learning (verbal memory), a digit-sequencing task (working memory), a token motor task (motor speed), category fluency and letter fluency (verbal fluency), symbol coding (attention and processing speed), and the Tower of London task (TOL; executive function). The primary scores for each BACS-J subtest were transformed into z-scores whereby the healthy control mean was set to zero and it^’^s standard deviation to one. An overall composite score for global cognition was generated by averaging all of the z-scores of the six BACS-J subtests for individual participants. In addition, the subjects were assessed with the Japanese version of the National Adult Reading Test (JART) [41-43] to measure the premorbid IQs of patients with schizophrenia [44], with reference to a previous report that estimated premorbid IQ with a Wide Range Achievement Test [36].

### Statistical analysis

SPSS for Windows (version 19.0; IBM Japan, Tokyo, Japan) was used for the statistical analysis. The differences in categorical variables (sex, smoking status, and prescription status of anxiolytics/hypnotics) between patients with schizophrenia and healthy controls or between the genotype groups were analyzed using a chi-square test. The demographic variables other than sex and smoking status, the clinical variables of schizophrenia other than prescription status of anxiolytics/hypnotics, and the BACS-J subtest scores were regarded as continuous variables and were assessed for normal distribution using a Shapiro–Wilk test (*P* < 0.05, implying a nonnormal distribution). The differences between the groups were analyzed with analyses of variance or with the nonparametric Kruskal–Wallis and Mann–Whitney *U*-tests if the variables showed a nonnormal distribution. Departure from the Hardy–Weinberg Equilibrium was analyzed for all SNPs using the exact test that was implemented in PLINK version 1.0.7 [45,46]. SNPs with *P* values less than 0.001 were considered to be in departure from the Hardy–Weinberg equilibrium, as described by Saito et al. [17]. The standard measures of pair-wise LD, denoted as *D*^*’*^ and r^2^, were estimated in Haploview 4.2 [47,48] based on the genotype data from all the participants.

Prior to the SNP- or haplotype-based association analyses that used general linear models, age, education years, JART-based premorbid IQ, and the BACS-J subtest scores, which showed nonnormal distributions, were standardized and normalized across all of the subjects with a mean of 0 and a variance of 1 using rank transformation according to Blom’s method [49,50]. Associations between each *HPS4* SNP and individual cognitive domains were analyzed using a linear regression model that was implemented in PLINK version 1.0.7 [45,46] with the covariates of age, sex, education years, and JART-based premorbid IQ because the BACS subtest performances were found to be affected by these confounding factors. The associations of each SNP with cognitive domains were tested under the three following genetic models that were fit for the minor alleles of each SNP: dominant (comparing minor allele carriers with major allele homozygotes), recessive (comparing minor allele homozygotes with major allele carriers), and additive (assuming an allelic dosage effect of minor alleles by coding none, one, or two copies of the minor allele as 0, 1, or 2, respectively). The associations of the haplotypes with individual cognitive domains were analyzed using haplotype-based association tests with a general linear model that was implemented in PLINK version 1.0.7 [45,46] with the same covariates as were entered into the single SNP-based association analysis. A sliding window approach with a window size of 2–5 SNPs was used for the analysis. Haplotype frequencies were computed using the standard expectation-maximization algorithm, and haplotypes with a frequency of greater than 5% in the whole sample were included in the analysis. A global (or omnibus) *P* value was calculated to jointly estimate all haplotype effects at the given locus. For both single SNP- and haplotype-based association analyses, *P* values were calculated by running 10,000 permutations using the max (T) procedure to correct for multiple testing between SNPs or haplotypes by each BACS-J subtest. A *P* value less than 0.0083 (0.05/6) was set as the threshold for significance in order to correct for the number of BACS-J subtests (six subtests) with Bonferroni correction. For haplotypes that met the significance criteria in the global test, a haplotype-specific test was performed to examine the independent effects of any estimated individual haplotypes by running 10,000 permutations in order to correct for multiple testing between haplotypes at the given locus.

### Power analysis

A power analysis for linear regression was calculated by G*Power 3.1.6 [51] using the Linear multiple regression: Fixed model, R^2^ deviation from zero procedure, which provided power analyses for testing the null hypothesis that the squared multiple correlation was zero [52,53]. Our cases and controls (n = 240, for each group) had an 80% power to detect an effect size of 0.08 or above at α = 0.0083.

## Results

### Association of HPS4 SNPs with cognitive domains

The detailed descriptive data for the patients with schizophrenia and the healthy controls are shown in Table 1. Both groups were well matched for age and sex. The patients with schizophrenia showed significantly lower education years (*P* < 0.001) and JART-based premorbid IQ (*P* < 0.001) and a significantly higher rate of current smoking (*P* = 0.015) than controls. They were found to have significantly lower performance in all cognitive domains as measured with the z-scores of the six BACS-J subtests (*P* < 0.001).

**Table 1:** Detailed descriptive data of patients with schizophrenia and healthy controls

	**Schizophrenia (n = 240)**	**Controls (n = 240)**	**z/*χ***^**2**^	***P*-value**
	**Mean (SD)**	**Mean (SD)**		
Age (years)	48.1 (12.4)	48.0 (13.0)	-0.03	0.98
Sex (male, %)^1^	57.9	59.6	0.14	0.71
Education (years)	11.7 (2.1)	14.2 (2.3)	-10.85	**<0.001**
Smokers (%)^1^	38.3	27.9	5.88	**0.015**
JART	92.4 (10.4)	103.4 (10.7)	-10.19	**<0.001**
Age at onset (years)	24.8 (8.9)	—	—	—
Antipsychotics (mg/day)^2^	12.7 (9.4)	—	—	—
Anticholinergics (mg/day)^3^	3.3 (4.2)	—	—	—
Anxiolytics/hypnotics (%)	75.4	—	—	—
PANSS				
Positive score	14.1 (5.3)	—	—	—
Negative score	21.9 (6.7)	—	—	—
General score	33.9 (9.5)	—	—	—
Total	69.0 (18.8)	—	—	—
BACS-J scores				
Verbal memory	-2.01 (1.31)	0.00 (1.00)	-14.63	**<0.001**
Working memory	-1.95 (1.22)	0.00 (1.00)	-14.67	**<0.001**
Motor speed	-1.48 (1.26)	0.00 (1.00)	-12.42	**<0.001**
Verbal fluency	-1.15 (1.14)	0.00 (1.00)	-10.62	**<0.001**
Attention	-1.97(1.14)	0.00 (1.00)	-15.41	**<0.001**
Executive function	-1.77 (2.26)	0.00 (1.00)	-9.67	**<0.001**
Composite Score	-1.72 (1.10)	0.00 (0.67)	-15.86	**<0.001**

*P*-values were analyzed using Mann–Whitney *U*-tests, except for ^1^***χ***^**2**^ tests. *P*-values < 0.05 are in bold.

^2^Haloperidol equivalent dosage.

^3^Biperiden equivalent dosage.

BACS-J: Brief Assessment of Cognition in Schizophrenia, Japanese-language version.

JART: Japanese version of the National Adult Reading Test.

PANSS: Positive and Negative Symptom Scale.

The characteristics of the selected five tagging SNPs (rs4822724, rs61276843, rs9608491, rs713998, and rs2014410) are shown in Table 2. All SNPs were in Hardy–Weinberg equilibrium (all *P* > 0.001), and they had minor allele frequencies greater than 10% in both groups. The standard LD measures, denoted as pair-wise D’ and r^2^ measures between markers in the *HPS4* gene, are illustrated in Additional file 1: Figure S1. For all SNPs, there were no significant differences in age, sex, education years, rates of current smoking, JART-based premorbid IQs, ages at onset, prescription status of anxiolytics/hypnotics, PANSS scores (positive scores, negative scores, general psychopathology scores, and total scores) between the genotype groups in the patients with schizophrenia (Additional file 2: Table S1 and Additional file 3: Table S2). The dosage of antipsychotics showed a significant difference between the rs9608491 genotype groups (*P* = 0.024), and the dosage of anticholinergics showed a significant difference between the rs713998 genotype groups (*P* = 0.031). The other SNP genotype groups showed no significant effects on the dosages of antipsychotics or anticholinergic drugs. In controls, there were no significant differences in any of the demographic characteristics (age, sex, education years, rates of current smoking, and JART scores) between the genotype groups of all SNPs (Additional file 4: Table S3).

**Table 2:** **Main characteristics of the selected tagging SNPs in *HPS4***

**No.**	**SNP (major/minor allele)**	**Localization (Residue Change)**		**HWE *P*-value**	**MAF**
1	rs4822724 (A/G)	5′-flanking region	Cases	0.0139	0.488
			Controls	0.2472	0.485
2	rs61276843^1^ (Del/Ins)	5′-flanking region	Cases	0.3959	0.133
			Controls	1.0000	0.121
3	rs9608491 (T/C)	intron 4	Cases	1.0000	0.179
			Controls	0.0569	0.188
4	rs713998 (G/A)	exon 9 (p.G229E)	Cases	0.1517	0.233
			Controls	0.8529	0.225
5	rs2014410 (C/G)	exon 11 (p.L443V)	Cases	0.1900	0.271
			Controls	0.0014	0.285

^1^21-base deletion/insertion (Del/Ins) polymorphism.

HWE: Hardy–Weinberg equilibrium test.

MAF: minor allele frequency.

The associations between each tagging SNP in *HPS4* and the cognitive domains that were measured by the BACS-J subtests in the patients with schizophrenia are shown in Table 3. Under the dominant model, rs713998 showed a significant association with executive function, and this association remained after correction for multiple testing by Bonferroni correction (β = 0.319, t = 3.191, permutation *P* value = 0.0073). Under this dominant model of rs713998, there was no significant difference in dosage of anticholinergics. The patients with a minor allele carrier (A/A and A/G = −1.21 ± 1.98, mean ± standard deviation) in rs713998 had a significantly higher z-score of executive function than patients with major allele homozygotes (G/G = −2.14 ± 2.36). In addition, rs713998 showed a nominally significant (*P* < 0.05) association with verbal memory, and this association did not remain after Bonferroni correction (verbal memory subtest score: A/A and A/G = −1.74 ± 1.28, G/G = −2.19 ± 1.31, β = 0.210, t = 2.704, permutation *P* value = 0.029). There was no significant difference between the *HPS4* SNPs and the cognitive domains under the recessive model or the additive model in the patients with schizophrenia.

**Table 3:** **Association of *HPS4 *** **SNPs with cognitive domains measured by the BACS-J in patients with schizophrenia**

**Cognitive domain**	**SNP**		**Dominant model**			**Recessive model**			**Additive model**									
		**β**	**t**	***P *** **(perm)**	**β**	**t**	***P *** **(perm)**	**β**	**t**	***P *** **(perm)**								
Verbal memory	rs4822724	-0.028	-0.296	0.999	-0.031	-0.323	0.998	-0.023	-0.388	0.991
	rs61276843	0.183	2.062	0.165	0.275	1.120	0.757	0.164	2.139	0.131
	rs9608491	-0.040	-0.489	0.989	0.147	0.644	0.967	-0.016	-0.226	0.999
	rs713998	0.210	2.704	**0.029**	0.097	0.644	0.967	0.146	2.386	0.074
	rs2014410	-0.128	-1.651	0.360	-0.236	-1.388	0.569	-0.123	-1.912	0.212
Working memory	rs4822724	0.183	1.882	0.243	-0.026	-0.255	1.000	0.065	1.037	0.746
	rs61276843	0.024	0.259	1.000	-0.123	-0.474	0.992	0.006	0.076	1.000
	rs9608491	0.117	1.370	0.554	0.254	1.063	0.797	0.115	1.535	0.406
	rs713998	0.068	0.824	0.896	-0.091	-0.580	0.982	0.026	0.408	0.988
	rs2014410	-0.012	-0.141	1.000	-0.313	-1.753	0.329	-0.053	-0.781	0.888
Motor speed	rs4822724	0.043	0.356	0.997	0.110	0.883	0.887	0.059	0.770	0.895
	rs61276843	-0.079	-0.685	0.951	0.038	0.118	1.000	-0.056	-0.555	0.969
	rs9608491	0.193	1.830	0.260	0.290	0.983	0.838	0.177	1.912	0.215
	rs713998	-0.081	-0.797	0.910	0.093	0.476	0.992	-0.035	-0.430	0.988
	rs2014410	0.069	0.683	0.952	-0.169	-0.763	0.936	0.024	0.280	0.998
Verbal fluency	rs4822724	-0.087	-0.680	0.947	-0.036	-0.272	1.000	-0.049	-0.601	0.956
	rs61276843	0.061	0.500	0.987	-0.066	-0.196	1.000	0.039	0.371	0.993
	rs9608491	0.047	0.421	0.993	0.026	0.083	1.000	0.039	0.394	0.991
	rs713998	0.152	1.412	0.524	0.210	1.016	0.820	0.129	1.530	0.419
	rs2014410	-0.050	-0.473	0.989	-0.102	-0.432	0.995	-0.050	-0.559	0.966
Attention	rs4822724	-0.075	-0.917	0.855	0.016	0.181	1.000	-0.025	-0.473	0.982
	rs61276843	0.065	0.815	0.903	0.099	0.453	0.994	0.058	0.847	0.858
	rs9608491	0.045	0.625	0.965	0.094	0.464	0.993	0.044	0.692	0.924
	rs713998	0.123	1.778	0.294	0.125	0.937	0.870	0.097	1.785	0.265
	rs2014410	-0.051	-0.738	0.932	-0.132	-0.871	0.897	-0.055	-0.948	0.803
Executive function	rs4822724	-0.132	-1.096	0.740	-0.132	-1.057	0.798	-0.104	-1.352	0.522
	rs61276843	0.257	2.242	0.111	-0.373	-1.169	0.730	0.156	1.561	0.390
	rs9608491	-0.054	-0.511	0.985	-0.150	-0.506	0.991	-0.056	-0.606	0.953
	rs713998	0.319	3.191	0.0073*	-0.050	-0.259	1.000	0.188	2.377	0.080
	rs2014410	-0.101	-1.002	0.797	-0.251	-1.133	0.752	-0.107	-1.269	0.578

Linear regression analyses were performed with the covariates of age, sex, education years, and JART scores. Permutation *P*-values, *P* (perm), were adjusted for multiple testing between SNPs by each BACS-J subtest *P*-values < 0.0083 (0.05/6) were considered as statistically significant to correct for the number of BACS-J subtests. *P*-values < 0.05 are in bold, and statistically significant results are marked with ‘*’.

BACS-J: Brief Assessment of Cognition in Schizophrenia, Japanese-language version.

In the controls (Table 4), rs9608491 and rs713998 were significantly associated with working memory under the recessive model, and these associations remained after correction for multiple testing with Bonferroni corrections (rs9608491, β = 0.701, t = 3.875, permutation *P* value = 0.001; rs713998, β = −0.646, t = −3.276, permutation *P* value = 0.0065). The healthy controls with minor homozygotes (C/C = 0.72 ± 0.84) in rs9608491 had a higher z-score of working memory than major allele carriers (T/T and C/T = −0.04 ± 0.99). Conversely, the healthy controls with minor homozygotes (A/A = −1.07 ± 0.96) in rs713998 had a lower z-score of working memory than major allele carriers (G/G and A/G = 0.05 ± 0.97). In addition, rs4822724 showed a nominally significant association with motor speed, and this association did not remain after Bonferroni correction (motor speed subtest score: G/G = 0.32 ± 0.97, A/A and A/G = −0.11 ± 0.99, β = 0.342, t = 3.027, permutation *P* value = 0.013). There was no significant difference between *HPS4* SNPs and cognitive domains under the dominant model in the healthy controls (*P* > 0.05). Under the additive model, the associations of working memory with the rs4822724, rs9608491, and rs713998 genotypes were nominally significant, and these associations did not remain after Bonferroni corrections on the additive model (z-scores of working memory subtest : rs4822724, G/G = 0.24 ± 0.93, A/G = 0.03 ± 0.97, A/A = −0.27 ± 1.06, β = 0.148, t = 2.574, permutation *P* value = 0.047; rs9608491, C/C = 0.72 ± 0.84, C/T = −0.06 ± 1.01, T/T = −0.04 ± 0.99, β = 0.209, t = 2.934, permutation *P* value = 0.017; rs713998, A/A = −1.07 ± 0.96, A/G = −0.08 ± 0.93, G/G = 0.13 ± 0.99, β = −0.217, t = −3.050, permutation *P* value = 0.011).

**Table 4:** **Association of *HPS4 *** **SNPs with cognitive domains measured by the BACS-J in healthy controls**

**Cognitive domain**	**SNP**	**Dominant model**			**Recessive model**			**Additive model**		
		**β**	**t**	***P *** **(perm)**	**β**	**t**	***P *** **(perm)**	**β**	**t**	***P *** **(perm)**
Verbal memory	rs4822724	0.075	0.870	0.873	0.147	1.670	0.380	0.081	1.539	0.402
	rs61276843	-0.071	-0.777	0.913	-0.072	-0.208	1.000	-0.065	-0.767	0.890
	rs9608491	0.029	0.352	0.997	0.303	1.798	0.304	0.064	0.975	0.778
	rs713998	-0.134	-1.724	0.317	-0.124	-0.675	0.967	-0.111	-1.700	0.310
	rs2014410	0.014	0.182	1.000	0.010	0.085	1.000	0.009	0.169	1.000
Working memory	rs4822724	0.225	2.412	0.069	0.174	1.793	0.313	0.148	2.574	**0.047**
	rs61276843	-0.150	-1.494	0.459	-0.882	-2.361	0.086	-0.181	-1.961	0.193
	rs9608491	0.162	1.797	0.283	0.701	3.875	0.001*	0.209	2.934	**0.017**
	rs713998	-0.187	-2.202	0.120	-0.646	-3.276	0.0065*	-0.217	-3.050	**0.011**
	rs2014410	-0.069	-0.824	0.893	0.096	0.747	0.945	-0.014	-0.236	0.999
Motor speed	rs4822724	0.085	0.764	0.921	0.342	3.027	**0.013**	0.155	2.274	0.095
	rs61276843	-0.179	-1.502	0.458	-0.280	-0.626	0.973	-0.169	-1.539	0.399
	rs9608491	0.045	0.417	0.994	0.159	0.722	0.951	0.052	0.613	0.951
	rs713998	-0.127	-1.259	0.627	-0.271	-1.139	0.749	-0.126	-1.475	0.439
	rs2014410	-0.090	-0.897	0.863	-0.075	-0.495	0.992	-0.062	-0.869	0.844
Verbal fluency	rs4822724	-0.048	-0.487	0.989	0.051	0.500	0.991	-7.1 × 10^-5^	-0.001	1.000
	rs61276843	-0.217	-2.084	0.168	-0.574	-1.469	0.522	-0.219	-2.287	0.092
	rs9608491	-0.149	-1.594	0.402	0.062	0.321	1.000	-0.086	-1.145	0.664
	rs713998	-0.171	-1.935	0.221	-0.160	-0.763	0.942	-0.143	-1.911	0.213
	rs2014410	0.174	1.993	0.199	0.051	0.379	0.998	0.099	1.590	0.373
Attention	rs4822724	0.090	1.171	0.697	0.063	0.793	0.929	0.057	1.199	0.629
	rs61276843	-0.141	-1.718	0.326	-0.359	-1.168	0.728	-0.141	-1.873	0.226
	rs9608491	0.062	0.842	0.892	0.229	1.508	0.481	0.074	1.257	0.585
	rs713998	-0.044	-0.629	0.962	-0.226	-1.378	0.575	-0.060	-1.026	0.744
	rs2014410	-0.008	-0.116	1.000	-0.035	-0.335	0.998	-0.004	-0.074	1.000
Executive function	rs4822724	0.003	0.032	1.000	0.039	0.381	0.997	0.015	0.248	0.998
	rs61276843	-0.049	-0.468	0.987	0.183	0.464	0.993	-0.031	-0.316	0.996
	rs9608491	0.003	0.035	1.000	-0.066	-0.339	0.998	-0.008	-0.104	1.000
	rs713998	0.020	0.229	1.000	0.099	0.470	0.993	0.027	0.363	0.993
	rs2014410	-0.049	-0.558	0.976	-0.057	-0.423	0.996	-0.037	-0.594	0.954

Linear regression analyses were performed with the covariates of age, sex, education years, and JART scores. Permutation *P*-values, *P* (perm), were adjusted for multiple testing between SNPs by each BACS-J subtest *P*-values < 0.0083 (0.05/6) were considered as statistically significant to correct for the number of BACS-J subtests. *P*-values < 0.05 are in bold, and statistically significant results are marked with ‘*’.

BACS-J: Brief Assessment of Cognition in Schizophrenia, Japanese-language version.

### Haplotype association analysis with cognitive functions

Additional file 5: Table S4 shows the global test scores for the associations of the 2–5 locus haplotypes constructed according to the sliding window approach with individual cognitive domains in the patients with schizophrenia and the healthy controls. When the five tagging SNPs (rs4822724, rs61276843, rs9608491, rs713998, and rs2014410) were denoted with serial SNP numbers (1, 2, 3, 4, and 5), haplotype 3–4 and 2-3-4 showed significant associations with working memory in the healthy controls, and these remained after correction for multiple testing with a Bonferroni correction (3–4: t = 14.9, permutation global *P* value = 0.0025; 2-3-4: t = 15.0, permutation global *P* value = 0.0064). The other haplotypes, except for haplotype 1–2, showed nominal associations with working memory (permutation global *P* value for each haplotype: 2–3, 0.017; 4–5, 0.012; 1-2-3, 0.030; 3-4-5, 0.011; 1-2-3-4, 0.018; 2-3-4-5, 0.013; 1-2-3-4-5, 0.031). The haplotype-specific tests of 3–4 and 2-3-4, which fulfilled the criteria for significance in the global haplotype test, with working memory were performed in the healthy controls (Table 5). In haplotype 3–4, T-A and C-G showed significant negative and positive effects, respectively, on working memory (T-A: β = −0.216, t = 9.30, permutation specific *P* value = 0.0074; C-G: β = 0.209, t = 8.61, permutation specific *P* value = 0.011). In haplotype 2-3-4, Del-C-G showed a significant positive effect on working memory (Del-C-G: β = 0.209, t = 8.61, permutation specific *P* value = 0.014). Del-C-G and C-G occurred at comparable frequencies, as did Del-T-G and T-G, indicating that haplotype 2-3-4 containing the major allele (G) at rs713998 was tagged most effectively by the major allele (del) at rs61276843. These associations remained significant after Bonferroni correction [*P* < 0.025 (0.05/2)] for multiple testing of the two classes (3–4 and 2-3-4) of the tested haplotypes.

**Table 5:** **Association of working memory with specific haplotypes of *HPS4 *** **in healthy controls**

**Haplotype**^**1**^		**Frequency**	**β**	**t**	**Specific *P *** **(perm)**
3-4	T-G	0.588	0.005	0.008	0.996
	T-A	0.225	-0.216	9.30	0.0074*
	C-G	0.187	0.209	8.61	0.011*
2-3-4	Del-T-G	0.585	0.006	0.012	0.999
	Del-C-G	0.187	0.209	8.61	0.014*
	Del-T-A	0.107	-0.194	4.11	0.141
	Ins-T-A	0.118	-0.180	3.75	0.171

Haplotype-specific tests with general linear models were performed with the covariates of age, sex, education years, and JART scores. Permutation specific *P*-values, specific *P* (perm), were adjusted for multiple testing between individual haplotypes at the given locus *P*-values < 0.025 (0.05/2) were considered as statistically significant to correct for the number of tested haplotypes (3–4 and 2-3-4). *P*-values < 0.05 are in bold, and statistically significant results are marked with ‘*’.

^1^SNP numbers are as follows: 2 = rs61276843, 3 = rs9608491, 4 = rs713998.

## Discussion

In the present study, which was based on our previous reports indicating an association of *HPS4* with schizophrenia [17], we evaluated the associations of *HPS4* with the clinical profiles of patients with schizophrenia and the cognitive functions of patients with schizophrenia and healthy controls. The major findings of our study were as follows: (1) the *HPS4* rs713998 polymorphism was significantly associated with executive function in Japanese patients with schizophrenia, and (2) the polymorphisms (rs9608491 and rs713998) and haplotypic variants (rs9608491-713998 and rs61276843-9608491-713998) in *HPS4* were significantly associated with working memory in Japanese healthy controls. These significances survived after corrections for multiple testing. Associations of the clinical profiles of patients with schizophrenia (PANSS scores, ages at onset, and premorbid IQs as measured by JART) with each SNP in *HPS4* were not observed.

Culminating lines of evidence have suggested that patients with schizophrenia show deficits of executive function, which is mainly mediated by the frontal lobe and which is involved in the ability of goal formation, planning, executing goal-directed plans, and effective performance [4,54]. The TOL task, which was employed as a task in order to assess executive function in the BACS [3], is known to be involved in multifaceted aspects of executive function, including working memory, planning, problem-solving, and inhibition [55,56]. Task complexity in performing the TOL correlated with activation of the dorsolateral prefrontal cortex, the lateral premotor cortex, the rostral anterior cingulate cortex, and the right dorsal caudate nucleus in a positron emission tomographic study of healthy subjects [57]. Moreover, decreased prefrontal activation during the TOL has been reported in subjects with first-episode schizophrenia [58]. We showed that the minor allele A that is carried at rs713998 was significantly associated with higher performance in executive function that was measured by the TOL task in patients with schizophrenia under the dominant genetic model, but there was no association between any of the *HPS4* SNPs and executive function in healthy controls. Because the sample size, age, and percentage of males were almost comparable between the patients with schizophrenia and the healthy controls, these results suggested that the rs713998 polymorphism confers distinct properties in frontal cortex-mediated executive function, specifically in patients with schizophrenia.

HS4 is unique in binding to the counterpart protein HPS1 and in forming a 1:1 heterodimer called BLOC-3 that is involved in late endosomal trafficking [14,15,33]. Both the N-terminal (amino acid: 1–274) and C-terminal domains (534–708) of HPS4 have been found to be required for assembly with a full-length HPS1 to form a soluble and stable BLOC-3 complex [34]. Rs713998 is a nonsynonymous SNP that is located in exon 9 (p.G229E) and that has been reported to be completely linked with three other nonsynonymous SNPs (rs5752330: exon 11, p.M552V; rs1894706: exon 12, p.Y606H; rs1894704: exon 13, p.H625G) of *HPS4*[17]. Given that the four SNPs (rs713998, rs5752330, rs1894706, and rs1894704) that completely link with each other occur in domains that are required for HPS1–HPS4 interaction (N-terminal domain: rs713998; C-terminal domain: rs5752330, rs1894706, and rs1894704), it is plausible that the amino-acid substitutions that are caused by these SNPs affect the HPS1–HPS4 interactions and BLOC-3 complex stability. The HPS1-HPS4 interaction is also indispensable for the recently identified function of BLOC-3 as a guanine nucleotide exchange factor (GEF) for the small GTPases Rab32/38 [35]. Although the longin domain that was bioinformatically predicted to occur in the N-terminal portion of HPS4 may be implicated in Rab GEF activity [35], no exonic tagging SNP corresponding to this domain was included in the present study.

Working memory impairments have been consistently reported in patients with schizophrenia, even those with first-episode schizophrenia with highly preserved IQ [59]. A digit-sequencing task, which has been employed as a task for assessing working memory in BACS [3], is akin to a number-letter sequencing task. The latter was found to activate the dorsolateral prefrontal cortex, the orbital frontal lobe, the premotor cortex, and the posterior parietal cortex, mainly in the right hemisphere, in a positron emission tomographic study of healthy subjects [60]. An SNP-based association analysis that was conducted under the recessive model and a haplotype-based association analysis have consistently shown that the C-allele at rs9608491 and the G-allele at 713998 conferred high performance in working memory in healthy controls. Rs9608491 would contribute to a greater proportion of the effect of this two-marker haplotype in the performance of working memory in this population due to its higher regression coefficient and smaller *P* value (β = 0.701, *P* value = 0.001) than rs713998 under the recessive model. However, the finding of the involvement of rs713998 in working memory is apparently paradoxical because rs713998 at the A-allele state exerts a beneficial effect on executive function in patients with schizophrenia. Thus, working memory and executive function that is measured by the TOL may reflect different aspects of cognition, even though they are intimately related to each other, and rs713998 may exert its effects on these cognitive subdomains across controls and patients. Moreover, the finding that two alleles carried at rs713998 showed differential effects on cognitive aspects in cases versus controls could indicate a kind of ceiling effect that cannot be accounted for by a simple dose–response relationship of the alleles. For example, it has been suggested that the catechol-O-methyltransferase Val158Met polymorphism has an opposite effect on verbal fluency in healthy controls compared with patients with schizophrenia [61]. In patients with schizophrenia, there has been no association of working memory with any polymorphism or haplotype that has been examined in *HPS4*, although medication and clinical symptoms may have the potential to obscure any effects of genetic variance. We previously found that protective haplotypes always contained the C-allele carried at rs9608491 in *HPS4* in a case–control study [17]. As discussed in our previous study [17], an argument for the involvement of variants of *HPS4*, particularly rs9608491, in the susceptibility to schizophrenia awaits further research. Collectively, the C-allele at rs9608491 may have a certain role in protecting against the susceptibility to schizophrenia and in enhancing working memory performance in controls only.

Very little information has thus far been found regarding the neurobiological and behavioral aspects of *HPS4*, although it is ubiquitously expressed in most tissues, including brain [11]. To the best of our knowledge, the Allen Brain Atlas [62] has only shown that HPS4 is expressed in the hippocampal formation, the olfactory areas, the cortical subplate, and the cerebellum of the male adult mouse using in situ hybridization. Notwithstanding the uncertainty about the role of this gene in these brain regions, the data obtained here regarding the association between rs9608491 and working memory suggested that *HPS4* plays a certain role in working memory that is driven by the appropriate function of the prefrontal cortex. Further analysis is required to reveal a putative role of *HPS4* in the neurobiology related to the cognitive deficits in schizophrenia.

Several limitations of the present study must be mentioned. First, although the total sample size was moderate for an association study of cognitive functions, a relatively small number of individuals were included as carriers of minor homozygotes for each SNP, except for rs4822724. This could have resulted in a selection bias and decreased the power under the recessive model when individuals carrying the minor homozygotes were assigned to a distinct group. The associations of working memory with rs9608491 and rs713998 in the healthy controls was revealed by the recessive model, which yielded a minor homozygote group with a small number of individuals (rs9608491 C/C, n = 13; rs713998 A/A, n = 11). Replication of these findings in an independent large cohort or in different ethnic cohorts is needed. Second, several lines of evidence have indicated that cognitive performance in patients with schizophrenia is influenced by psychopharmacological medications [63,64]. In our positive finding of an association between rs713998 and executive function in patients with schizophrenia, we confirmed there was no significant difference (*P* > 0.05) in the dosages of antipsychotics, anticholinergics, or the prescription status of anxiolytics/hypnotics under the dominant model. However, in a real-world clinical setting, the extent to which various kinds of antipsychotics or their combination, particularly in the context of the polypharmacy of antipsychotic medication, exerts an effect, if any, on the cognitive performance of patents with schizophrenia is difficult to analyze. Third, rs9608491 is an intronic SNP located in between the exons encoding the N-terminal domain. Its function is uncertain because it is unlikely to affect the stability of HPS4 mRNA [17]. This is reminiscent of the relevant SNPs of the *DTNBP1* gene, which are mostly intronic and have been significantly implicated in the executive function of healthy controls [30], in cases belonging to early-onset families with functional psychosis disorders [65], and in the spatial working memory in patients with schizophrenia [66]. This general aspect regarding the question as to why intronic SNPs are involved in cognitive function awaits further genetic study.

## Conclusions

Our present study demonstrated that genetic variants in *HPS4* were associated with executive function in patients with schizophrenia and with working memory in healthy controls. To the best of our knowledge, this is the first report of an association of *HPS4* with cognitive functions and the first to employ the BACS-J as a battery in a SNP- and haplotype-based association study of cognitive functions. Our present results suggested that *HPS4* may be involved in the executive function deficits of patients with schizophrenia. Further research is needed to elucidate the role of *HPS4* in higher brain functions through neurobiological and behavioral analyses of animal models.

## Competing interests

Kazufumi Akiyama is a paid consultant to Taisho Toyama Pharmaceutical Co., Ltd. This consultancy had no further role in the study design, the collection, analysis and interpretation of data, the writing of the report, or the decision to submit the paper for publication. None of the remaining authors declare any conflicts of interest.

## Authors’ contributions

GK was involved in the design of the study, performed the statistical analysis, and drafted the manuscript. AS was involved in the design of the study, sample collection, and genotyping. YO, TW, KF, KS, TI, HM and KO were involved in participation in the design of the study and coordination for sample collection. KA was involved in the design of the study, laboratory coordination, sample collection, and editing the manuscript. All authors read and approved final manuscript.

## Pre-publication history

The pre-publication history for this paper can be accessed here:

http://www.biomedcentral.com/1471-244X/13/276/prepub

## Supplementary Material

Additional file 1: Figure S1 Linkage disequilibrium (LD) block structure of the *HPS4* gene. The values in the boxes represent the pair-wise *D*^*’*^and r^2^ measures between markers. Boxes without values indicate ∣*D’*-value∣ = 1.00. Click here for file

Additional file 2: Table S1 Demographics for each genotype group of *HPS4* SNPs in patients with schizophrenia. Click here for file

Additional file 3: Table S2 Clinical characteristics for each genotype group of *HPS4* SNPs in patients with schizophrenia. Click here for file

Additional file 4: Table S3 Demographics for each genotype group of *HPS4* SNPs in healthy controls. Click here for file

Additional file 5: Table S4 Global haplotype association for 2–5 SNPs in *HPS4* with cognitive domains in patients and controls. Click here for file
