# Supplementary figures and images for: Association of the Hermansky–Pudlak syndrome type 4 (HPS4) gene variants with cognitive function in patients with schizophrenia and healthy subjects
Source: BMC Psychiatry. 2013 Oct 30;13:276. doi: 10.1186/1471-244X-13-276 (PMC3819706; doi:10.1186/1471-244X-13-276)

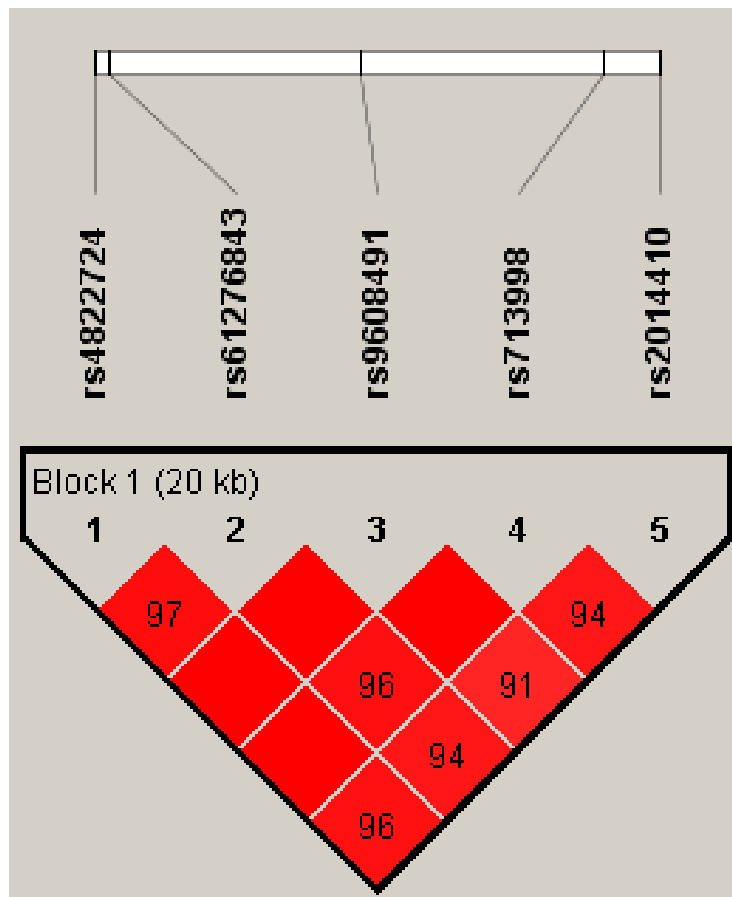

**$D'$**

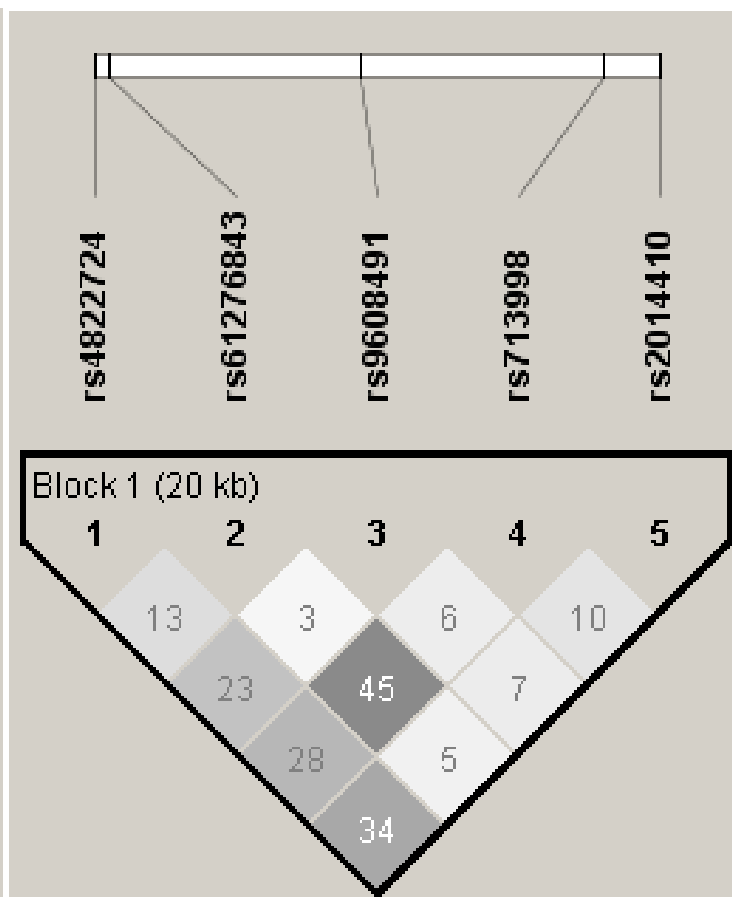

**$r^2$**

Supplement: Additional file 1: Figure S1 — Linkage disequilibrium (LD) block structure of the HPS4 gene. The values in the boxes represent the pair-wise D’and r2 measures between markers. Boxes without values indicate ∣D’-value∣ = 1.00. [file 1471-244X-13-276-S1.pdf]
