# Supplementary material for: Association of the Hermansky–Pudlak syndrome type 4 (HPS4) gene variants with cognitive function in patients with schizophrenia and healthy subjects
Source: BMC Psychiatry. 2013 Oct 30;13:276. doi: 10.1186/1471-244X-13-276 (PMC3819706; doi:10.1186/1471-244X-13-276)
Supplement: Additional file 2: Table S1 — Demographics for each genotype group of HPS4 SNPs in patients with schizophrenia. [file 1471-244X-13-276-S2.doc]

**Table S1 Demographics for each genotype group of *HPS4* SNPs in patients with schizophrenia**

| **SNP** | **Genotype** | **n** | **Age**  **[years]** | **Sex^1^**  **[male, %]** | **Education**  **[years]** | **Smokers^1^**  **[%]** | **JART** |
| --- | --- | --- | --- | --- | --- | --- | --- |
| rs4822724 | A/A | 53 | 47.1 (11.2) | 67.9 | 12.0 (2.2) | 41.5 | 91.5 (9.4) |
|  | A/G | 140 | 48.4 (13.0) | 52.9 | 11.5 (2.0) | 39.3 | 92.4 (10.2) |
|  | G/G | 47 | 48.2 (12.1) | 61.7 | 12.0 (2.2) | 31.9 | 93.7 (12.1) |
| rs61276843 | Del/Del | 182 | 48.2 (12.7) | 59.9 | 11.7 (2.1) | 38.5 | 92.5 (10.7) |
|  | Del/Ins | 52 | 48.0 (12.1) | 53.8 | 11.5 (1.9) | 38.5 | 92.3 (9.7) |
|  | Ins/Ins | 6 | 46.3 (6.4) | 33.3 | 12.8 (2.4) | 33.3 | 90.8 (9.2) |
| rs9608491 | T/T | 161 | 47.4 (12.7) | 57.8 | 11.8 (2.2) | 40.4 | 92.0 (10.0) |
|  | C/T | 72 | 49.3 (11.9) | 56.9 | 11.4 (1.9) | 33.3 | 93.0 (11.3) |
|  | C/C | 7 | 50.3 (11.9) | 71.4 | 12.4 (2.1) | 42.9 | 95.5 (13.1) |
| rs713998 | G/G | 145 | 49.2 (12.6) | 59.3 | 11.6 (2.1) | 36.6 | 92.5 (11.4) |
|  | A/G | 78 | 47.1 (12.4) | 56.4 | 11.7 (2.1) | 41.0 | 92.4 (8.7) |
|  | A/A | 17 | 43.4 (10.8) | 52.9 | 12.4 (2.2) | 41.2 | 91.9 (10.0) |
| rs2014410 | C/C | 123 | 46.6 (12.4) | 55.3 | 12.0 (2.1) | 33.3 | 93.4 (10.5) |
|  | C/G | 104 | 50.1 (12.3) | 57.7 | 11.3 (1.9) | 45.2 | 91.7 (10.5) |
|  | G/G | 13 | 46.1 (12.2) | 84.6 | 11.8 (2.7) | 30.8 | 89.2 (9.2) |

Mean (SD). For all SNPs, there were no significant differences (*P* > 0.05) in all demographic characteristics between genotype groups (Kruskal–Wallis tests or ^1^χ^2^ tests).

JART: Japanese version of the National Adult Reading Test
