# Supplementary material for: Association of the Hermansky–Pudlak syndrome type 4 (HPS4) gene variants with cognitive function in patients with schizophrenia and healthy subjects
Source: BMC Psychiatry. 2013 Oct 30;13:276. doi: 10.1186/1471-244X-13-276 (PMC3819706; doi:10.1186/1471-244X-13-276)
Supplement: Additional file 3: Table S2 — Clinical characteristics for each genotype group of HPS4 SNPs in patients with schizophrenia. [file 1471-244X-13-276-S3.doc]

**Table S2 Clinical characteristics for each genotype group of *HPS4* SNPs in patients with schizophrenia**

| **SNP** | **Genotype** | **n** | **Onset**  **[years]** | **Medication** | | |  | **PANSS** | | | |
| --- | --- | --- | --- | --- | --- | --- | --- | --- | --- | --- | --- |
|  |  |  |  | **Anti-**  **psychotics^3^**  **[mg/day]** | **Anti-**  **cholinergics^4^**  **[mg/day]** | **Anxiolytics/**  **Hypnotics^2^**  **[%]** |  | **Positive**  **score** | **Negative**  **score** | **General**  **score** | **Total**  **score** |
| **rs4822724** | A/A | 53 | 23.3 (7.1) | 12.4 (8.1) | 3.6 (4.6) | 69.8 |  | 13.9 (4.9) | 21.9 (6.2) | 33.0 (9.4) | 68.7^1^ (17.6) |
|  | A/G | 140 | 25.7 (9.5) | 12.6 (9.3) | 3.0 (3.5) | 78.6 |  | 14.2 (5.6) | 22.0 (7.4) | 33.2 (9.8) | 69.3^1^ (20.0) |
|  | G/G | 47 | 23.9 (8.4) | 13.5 (11.0) | 3.6 (5.5) | 72.3 |  | 14.1 (5.0) | 21.8 (5.3) | 32.0 (9.0) | 69.0^1^ (18.8) |
| **rs61276843** | Del/Del | 182 | 24.7 (8.9) | 12.9 (9.4) | 3.4 (4.5) | 76.9 |  | 14.1 (5.4) | 21.9^1^ (6.8) | 32.9 (9.5) | 68.9^1^ (19.0) |
|  | Del/Ins | 52 | 25.3 (9.2) | 12.4 (9.5) | 3.0 (3.2) | 75.0 |  | 14.0 (5.1) | 22.0^1^ (6.6) | 33.0 (9.8) | 69.1^1^ (18.3) |
|  | Ins/Ins | 6 | 24.0 (6.5) | 9.8 (6.0) | 1.7 (1.4) | 33.3 |  | 15.0 (5.4) | 23.2^1^ (7.7) | 32.0 (10.3) | 70.2^1^ (20.7) |
| **rs9608491** | T/T | 161 | 24.6 (8.5) | **12.6 (8.8)** | 3.5 (4.7) | 75.2 |  | 14.1 (5.4) | 22.2^1^ (6.9) | 33.3 (9.3) | 69.6^1^ (18.7) |
|  | C/T | 72 | 25.4 (9.8) | **13.6 (10.6)** | 2.9 (3.1) | 79.2 |  | 14.2 (5.4) | 21.4^1^ (6.7) | 32.1 (10.1) | 67.7^1^ (19.6) |
|  | C/C | 7 | 23.6 (6.4) | **5.3 (3.8)** | 1.1 (1.2) | 42.9 |  | 13.4 (4.4) | 21.4^1^ (3.6) | 31.1 (9.5) | 66.0^1^ (15.0) |
| **rs713998** | G/G | 145 | 24.7 (9.1) | 12.5 (9.2) | **3.6 (4.5)** | 75.9 |  | 13.9 (5.5) | 22.1 (6.9) | 32.5 (9.4) | 68.6^1^ (19.1) |
|  | A/G | 78 | 25.5 (9.0) | 13.0 (10.2) | **2.4 (3.5)** | 76.9 |  | 14.2 (5.2) | 21.7 (6.7) | 33.4 (10.2) | 69.3^1^ (19.2) |
|  | A/A | 17 | 22.2 (5.7) | 13.1 (7.1) | **4.3 (4.0)** | 64.7 |  | 15.4 (4.7) | 21.6 (5.7) | 33.2 (7.8) | 70.1^1^ (15.1) |
| **rs2014410** | C/C | 123 | 24.6 (8.5) | 13.6 (10.2) | 3.1 (4.1) | 74.0 |  | 14.6 (5.4) | 21.6 (6.2) | 32.9 (9.7) | 69.2 (18.5) |
|  | C/G | 104 | 25.5 (9.5) | 11.5 (8.4) | 3.3 (4.4) | 76.9 |  | 13.7 (5.2) | 22.3 (7.4) | 32.7 (9.3) | 68.7 (19.1) |
|  | G/G | 13 | 21.0 (4.6) | 14.4 (7.9) | 4.4 (3.7) | 76.9 |  | 13.3 (6.2) | 22.5 (6.3) | 33.6 (10.7) | 69.5 (20.8) |

Mean (SD). For all SNPs, clinical characteristics between genotype groups were analyzed using Kruskal-Wallis tests, except for ^1^analyses of variance (normally distributed variables) and ^2^χ^2^ tests (categorical variables). *P*-values < 0.05 are in bold.

^3^Haloperidol equivalent dosage.

^4^Biperiden equivalent dosage.

PANSS: Positive and Negative Symptom Scale
