# Supplementary material for: Association of the Hermansky–Pudlak syndrome type 4 (HPS4) gene variants with cognitive function in patients with schizophrenia and healthy subjects
Source: BMC Psychiatry. 2013 Oct 30;13:276. doi: 10.1186/1471-244X-13-276 (PMC3819706; doi:10.1186/1471-244X-13-276)
Supplement: Additional file 4: Table S3 — Demographics for each genotype group of HPS4 SNPs in healthy controls. [file 1471-244X-13-276-S4.doc]

**Table S3 Demographics for each genotype group of *HPS4* SNPs in healthy controls**

| **SNP** | **Genotype** | **n** | **Age**  **[years]** | **Sex^1^**  **[male, %]** | **Education**  **[years]** | **Smokers^1^**  **[%]** | **JART** |
| --- | --- | --- | --- | --- | --- | --- | --- |
| rs4822724 | A/A | 68 | 49.2 (13.1) | 50.0 | 14.1 (2.4) | 22.1 | 104.8 (11.5) |
|  | A/G | 111 | 48.0 (13.3) | 60.4 | 14.3 (2.4) | 31.5 | 102.9 (10.8) |
|  | G/G | 61 | 46.7 (12.4) | 68.9 | 14.2 (2.1) | 27.9 | 102.7 (9.6) |
| rs61276843 | Del/Del | 185 | 47.5 (12.9) | 61.1 | 14.4 (2.1) | 27.6 | 103.3 (10.0) |
|  | Del/Ins | 52 | 49.8 (13.2) | 53.8 | 13.6 (2.8) | 28.8 | 103.7(12.8) |
|  | Ins/Ins | 3 | 51.3 (14.5) | 66.7 | 13.0 (3.6) | 33.3 | 101.0 (17.4) |
| rs9608491 | T/T | 163 | 47.9 (12.8) | 59.5 | 14.3 (2.4) | 25.2 | 104.3 (11.0) |
|  | C/T | 64 | 48.5 (13.7) | 60.9 | 14.2 (2.1) | 34.4 | 101.7 (9.9) |
|  | C/C | 13 | 46.9 (13.1) | 53.8 | 13.3 (2.1) | 30.8 | 100.8 (10.7) |
| rs713998 | G/G | 143 | 47.5 (12.8) | 62.2 | 14.3 (2.1) | 27.3 | 103.7 (10.0) |
|  | A/G | 86 | 48.4 (13.4) | 58.1 | 14.1 (2.6) | 30.2 | 103.0 (11.9) |
|  | A/A | 11 | 51.1 (13.3) | 36.4 | 13.4 (2.8) | 18.2 | 103.0 (11.4) |
| rs2014410 | C/C | 133 | 48.0 (12.9) | 62.4 | 14.1 (2.4) | 30.1 | 103.0 (10.6) |
|  | C/G | 77 | 47.7 (13.4) | 57.1 | 14.2 (2.3) | 27.3 | 102.7 (11.1) |
|  | G/G | 30 | 48.8 (12.9) | 53.3 | 14.8 (2.0) | 20.0 | 106.8 (10.1) |

Mean (SD). For all SNPs, there were no significant differences (*P* > 0.05) in all demographic characteristics between genotype groups (Kruskal–Wallis tests or ^1^χ^2^ tests).

JART: Japanese version of the National Adult Reading Test
