# Supplementary material for: Association of the Hermansky–Pudlak syndrome type 4 (HPS4) gene variants with cognitive function in patients with schizophrenia and healthy subjects
Source: BMC Psychiatry. 2013 Oct 30;13:276. doi: 10.1186/1471-244X-13-276 (PMC3819706; doi:10.1186/1471-244X-13-276)
Supplement: Additional file 5: Table S4 — Global haplotype association for 2–5 SNPs in HPS4 with cognitive domains in patients and controls. [file 1471-244X-13-276-S5.doc]

**Table S4 Global haplotype association for 2–5 SNPs in *HPS4* with cognitive domains in patients and controls**

|  | **Haplotype^1^** | **Verbal**  **memory** | |  | **Working**  **memory** | |  | **Motor**  **speed** | |  | **Verbal**  **fluency** | |  | **Attention** | |  | **Executive**  **function** | |
| --- | --- | --- | --- | --- | --- | --- | --- | --- | --- | --- | --- | --- | --- | --- | --- | --- | --- | --- |
|  |  | **t** | ***P***  **(perm)** |  | **t** | ***P***  **(perm)** |  | **t** | ***P***  **(perm)** |  | **t** | ***P***  **(perm)** |  | **t** | ***P***  **(perm)** |  | **t** | ***P***  **(perm)** |
| Patients |  |  |  |  |  |  |  |  |  |  |  |  |  |  |  |  |  |  |
|  | 1-2 | 4.79 | 0.236 |  | 1.34 | 0.810 |  | 0.67 | 0.940 |  | 0.38 | 0.980 |  | 0.74 | 0.929 |  | 3.09 | 0.452 |
|  | 2-3 | 4.60 | 0.257 |  | 2.50 | 0.563 |  | 3.67 | 0.370 |  | 0.37 | 0.982 |  | 1.49 | 0.775 |  | 2.52 | 0.551 |
|  | 3-4 | 5.82 | 0.155 |  | 3.02 | 0.470 |  | 3.64 | 0.374 |  | 2.98 | 0.478 |  | 4.59 | 0.258 |  | 5.62 | 0.170 |
|  | 4-5 | 6.95 | 0.099 |  | 0.68 | 0.936 |  | 0.19 | 0.995 |  | 2.34 | 0.600 |  | 3.21 | 0.439 |  | 5.70 | 0.164 |
|  | 1-2-3 | 4.77 | 0.419 |  | 2.77 | 0.735 |  | 3.70 | 0.576 |  | 0.97 | 0.975 |  | 1.84 | 0.878 |  | 3.08 | 0.664 |
|  | 2-3-4 | 5.57 | 0.320 |  | 2.95 | 0.705 |  | 3.66 | 0.583 |  | 3.30 | 0.649 |  | 3.83 | 0.554 |  | 5.93 | 0.280 |
|  | 3-4-5 | 6.83 | 0.208 |  | 3.23 | 0.651 |  | 4.63 | 0.439 |  | 3.20 | 0.665 |  | 4.66 | 0.437 |  | 6.09 | 0.265 |
|  | 1-2-3-4 | 7.39 | 0.287 |  | 3.46 | 0.785 |  | 2.77 | 0.871 |  | 3.37 | 0.801 |  | 4.06 | 0.696 |  | 5.70 | 0.465 |
|  | 2-3-4-5 | 6.76 | 0.345 |  | 2.66 | 0.884 |  | 3.59 | 0.768 |  | 2.32 | 0.808 |  | 3.90 | 0.719 |  | 5.65 | 0.473 |
|  | 1-2-3-4-5 | 7.50 | 0.411 |  | 6.06 | 0.581 |  | 3.80 | 0.858 |  | 3.76 | 0.869 |  | 4.08 | 0.830 |  | 6.15 | 0.562 |
| Controls |  |  |  |  |  |  |  |  |  |  |  |  |  |  |  |  |  |  |
|  | 1-2 | 2.53 | 0.533 |  | 8.14 | 0.054 |  | 6.72 | 0.097 |  | 6.86 | 0.091 |  | 4.21 | 0.279 |  | 0.08 | 0.999 |
|  | 2-3 | 1.29 | 0.794 |  | 10.7 | **0.017** |  | 2.45 | 0.543 |  | 8.02 | 0.055 |  | 4.32 | 0.266 |  | 0.13 | 0.997 |
|  | 3-4 | 3.25 | 0.408 |  | 14.9 | **0.0025*** |  | 2.25 | 0.583 |  | 6.35 | 0.113 |  | 2.16 | 0.609 |  | 0.13 | 0.997 |
|  | 4-5 | 3.14 | 0.426 |  | 11.6 | **0.012** |  | 4.57 | 0.238 |  | 4.54 | 0.244 |  | 1.30 | 0.800 |  | 0.37 | 0.976 |
|  | 1-2-3 | 2.65 | 0.722 |  | 11.6 | **0.030** |  | 6.82 | 0.191 |  | 8.71 | 0.094 |  | 4.83 | 0.387 |  | 0.21 | 1.000 |
|  | 2-3-4 | 3.21 | 0.629 |  | 15.0 | **0.0064*** |  | 2.49 | 0.750 |  | 8.28 | 0.109 |  | 4.56 | 0.427 |  | 0.54 | 0.994 |
|  | 3-4-5 | 3.62 | 0.563 |  | 14.1 | **0.011** |  | 4.79 | 0.391 |  | 5.93 | 0.267 |  | 2.25 | 0.799 |  | 0.45 | 0.996 |
|  | 1-2-3-4 | 3.91 | 0.690 |  | 14.8 | **0.018** |  | 6.90 | 0.311 |  | 8.99 | 0.157 |  | 5.09 | 0.529 |  | 0.82 | 0.997 |
|  | 2-3-4-5 | 3.35 | 0.774 |  | 15.8 | **0.013** |  | 4.60 | 0.589 |  | 8.59 | 0.180 |  | 3.98 | 0.687 |  | 0.98 | 0.994 |
|  | 1-2-3-4-5 | 3.78 | 0.710 |  | 13.4 | **0.031** |  | 6.27 | 0.378 |  | 9.19 | 0.146 |  | 4.37 | 0.628 |  | 0.96 | 0.994 |

Haplotype-based association analyses with general linear models were performed with the covariates of age, sex, education years, and JART scores. Permutation global *P*-values, *P* (perm), were adjusted for multiple testing between haplotypes by each BACS-J subtest *P*-values < 0.0083 (0.05/6) were considered as statistically significant to correct for the number of BACS-J subtests. *P*-values < 0.05 are in bold, and statistically significant results are marked with '*'.

^1^SNP numbers are as follows: 1 = rs4822724; 2 = rs61276843, 3 = rs9608491, 4 = rs713998, 5 = rs2014410.

BACS-J: Brief Assessment of Cognition in Schizophrenia, Japanese-language version.
